# Alu–based cell-free DNA: a novel biomarker for screening of gastric cancer

**DOI:** 10.18632/oncotarget.11079

**Published:** 2016-08-05

**Authors:** Chen Qian, Shaoqing Ju, Jing Qi, Jianmei Zhao, Xianjuan Shen, Rongrong Jing, Juan Yu, Li Li, Yingjuan Shi, Lurong Zhang, Zhiwei Wang, Hui Cong

**Affiliations:** ^1^ Center of Laboratory Medicine, Affiliated Hospital of Nantong University, Nantong, Jiangsu 226001, China; ^2^ Surgical Comprehensive Laboratory, Affiliated Hospital of Nantong University, Nantong, Jiangsu 226001, China; ^3^ Department of Pediatrics, Affiliated Hospital of Nantong University, Nantong, Jiangsu 226001, China; ^4^ Department of Radiation Oncology, UF Shands Cancer Center, University of Florida, Gainesville, FL 32610, USA; ^5^ Department of General Surgery, Affiliated Hospital of Nantong University, Nantong, Jiangsu 226001, China

**Keywords:** branched DNA assay, cell-free DNA, Alu sequence, gastric cancer, serum

## Abstract

Gastric cancer (GC) is the fourth most common cancer and the second major cause of cancer-related deaths worldwide. In our previous study, a novel and sensitive method for quantifying cell-free DNA (CFD) in human blood was established and tested for its ability to predict patients with tumor. We want to investigate CFD expression in the sera of GC patients in an attempt to explore the clinical significance of CFD in improving the early screening of GC and monitoring GC progression by the branched DNA (bDNA)-based *Alu* assay. The concentration of CFD was quantitated by bDNA-based *Alu* assay. CEA, CA19-9, C72-4 and CA50 concentrations were determined by ABBOTT ARCHITECT I2000 SR. We found the CFD concentrations have significant differences between GC patients, benign gastric disease (BGD) patients and healthy controls (*P* < 0.05). CFD were weakly correlated with CEA (*r* = −0.197, *P* < 0.05) or CA50 (*r* = 0.206, *P* < 0.05), and no correlation with CA19-9 (*r* = −0.061, *P* > 0.05) or CA72-4 (*r* = 0.011, *P* > 0.05). In addition, CFD concentrations were significantly higher in stage I GC patients than BGD patients and healthy controls (*P* < 0.05), but there was no significant difference in CEA, CA19-9 and CA50 among the three traditional tumor markers (*P* > 0.05). Our analysis showed that CFD was more sensitive than CEA, CA19-9, CA72-4 or CA50 in early screening of GC. Compared with CEA, CA19-9, CA72-4 and CA50, CFD may prove to be a better biomarker for the screening of GC, thus providing a sensitive biomarker for screening and monitoring progression of GC.

## INTRODUCTION

Advances in molecular diagnosis and therapeutic approaches for gastric cancer (GC) have decreased the mortality rates of GC patients in recent years, but it is still remains the fourth most common cancer and the second major cause of cancer-related deaths worldwide [1–4]. The symptoms of GC and precancerous lesions are often occult and non-specific, and only 5–10% GC patients can be diagnosed in the early stage in China. Gastroscopy and iconography are mostly used for early diagnosis of GC at present, but the problems of invasiveness, high cost and the possible infliction of pain on the patient limit their use for early screening in a wide range of patients. Although tumor-associated antigens on the surface of GC cells, including CEA, CA72-4 and CA50, can be used as indicators for early screening of GC, they are handicapped by high false positive and negative rates, low specificity and sensitivity. Therefore, a combined detection system is needed [5], and researchers have made every effort in an attempt to find a minimally traumatic, sensitive, reliable and GC-specific tumor marker to improve early screening.

Cell-free DNA (CFD) is a cell-free status of extracellular DNA existing in the blood (serum or plasma), synovial fluid, cerebrospinal fluid (CSF) and other body fluids. There are various methods for quantitative CFD detection, but their efficiency is limited to the sample preparation, which greatly limits the research of free DNA. In our previous study, we used an *Alu* sequence-based sensitive branched DNA quantitative method to detect CFD levels, knowing that the branched DNA (bDNA) technology is a signal amplification technique that can be used for quantitative detection of free DNA, RNA and miRNA [6]. Compared with real-time PCR, the new method improved the detection sensitivity by increasing the labeled probe copy number or signal intensity of the marker without amplifying the target sequences, thus overcoming the false positive rate of PCR detection [7–9]. In 2006, Umetani et al. found that tumor-associated cell-free DNA in serum was positively correlated with disease progression and tumor load, and could be expressed by *Alu*247/*Alu*115 ratio [10]. Therefore, more CFD of *Alu* repeat sequences could be detected in serum, which theoretically has more universal significance.

In this study, we were aimed to assess the diagnostic value of *Alu*-based serum CFD in GC patients by bDNA, explore the possible correlation between serum CFD and GC-associated tumor markers of CEA, CA19-9, CA72-4 and CA50, and evaluate the assistant diagnostic value of serum CFD with bDNA technology in early screening and monitoring progression.

## RESULTS

### Serum CFD discriminates between patients with GC or benign gastric disease and healthy controls

**Figure 1:**
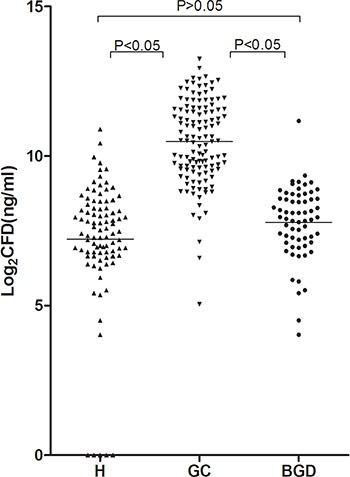
CFD concentrations in GC patients and healthy controls Mann Whitney test was used in this figure. CFD levels were measured in 124 unselected GC patients, 64 unselected benign gastric disease patients (gastric adenoma patients)and 92 healthy controls. The results for the CFD levels are presented as the median with the 25th and the 75th percentile values. Horizontal lines indicate the median for each group. CFD: cell-free DNA; GC: gastric cancer; BGD: benign gastric disease; H: healthy controls.

The concentration of CFD was measured using the *Alu*-based bDNA assay in GC patients, BGD patients, and healthy controls. The median CFD concentration was significantly higher in GC patients (1475.92 ng/ml, range: 774.36–3059.69 ng/ml) than that in BGD patients (244.42 ng/ml, range: 141. 18–385.74 ng/ml, *P* < 0.05) and healthy controls (181.90 ng/ml, range: 109.50–328.68 ng/ml, *P* < 0.05). There was no statistical difference between BGD patients and healthy controls (*P* > 0.05) (Figure 1).

### Serum CFD levels in GC patients with different clinicopathological characteristics

The association between the CFD concentration and various clinicopathological parameters such as age, gender, tumor location and tumor size was investigated. As evidenced from the data in Table 1, no significant association was found between the CFD concentration and the patient age, gender and tumor location (*P* > 0.05). However, the CFD concentration in patients with tumor size > 5 cm were significantly higher than those with tumor size < 5 cm (*P* < 0.05).

**Table 1:** Relationship between CFD expression level and patient clinicopathological characteristics

Parameters	*n*	Median(range) ng/ml	*P*
Age (y)			> 0.05
≤ 60	52	1515.55 (682.41–3574.67)	
> 60	72	1463.02 (806.03–3043.20)	
Gender			> 0.05
M	89	1404.82 (724.78–3156.99)	
F	35	1975.70 (906.49–2675.39)	
Location			> 0.05
Upper	23	1130.26 (641.66–2578.35)	
Medium	49	1053.86 (666.60–3117.14)	
Lower	52	2076.04 (909.33–3203.02)	
Tumor size			< 0.05
≤ 5 cm	48	1039.94 (485.60–3911.31)	
> 5 cm	76	3046.52 (1131.74–4138.82)	

### Serum CFD levels are correlated with GC clinical stages

**Figure 2:**
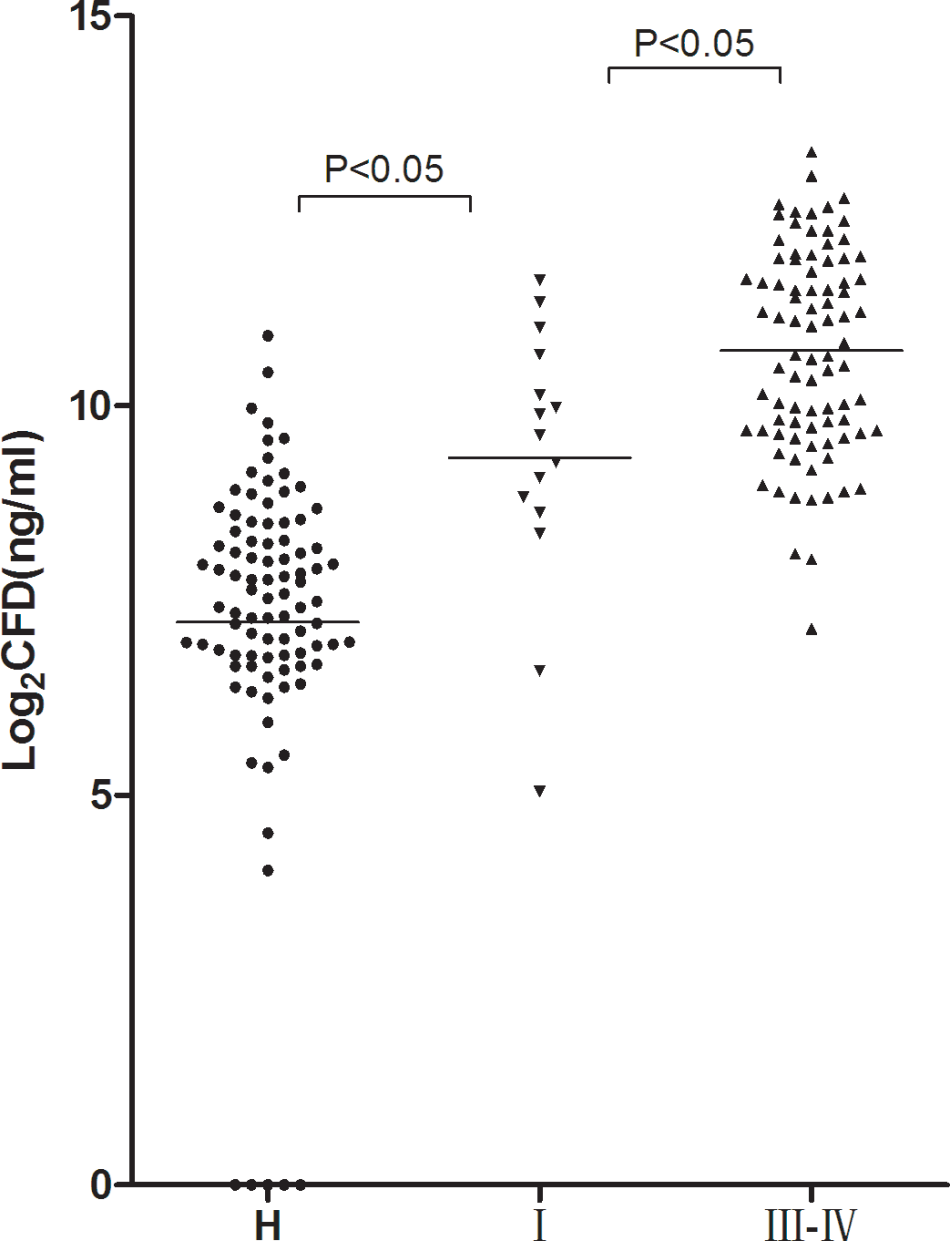
CFD concentrations in GC patients with different clinical stages and healthy controls . Mann Whitney test was used in this figure. CFD levels were measured in 15 stage I GC patients, 86 stage III ˜ IV GC patients, and 92 healthy controls. The results for the CFD levels are presented as the median with the 25th and the 75th percentile values. Horizontal lines indicate the median for each group. CFD: cell-free DNA; H: healthy controls; I: stage I GC patients; III ˜ IV: stage III ˜ IV GC patients.

The concentrations of CFD in stage I, II and III ˜ IV GC patients were 785.46 ng/ml (395.05–1616.85 ng/ml), 1483.01 ng/ml (747.08–2430.86 ng/ml) and 1700.26 ng/ml (820.94–3739.02 ng/ml) respectively. It was found that the mean serum CFD level in stage III ˜ IV GC patients was significantly higher than that in stage I GC patients (*P* < 0.05) (Table 2). Serum CFD level of stage I GC patients was about 6 times that in healthy controls. There was a significantly difference between the two groups (*P* < 0.05) (Figure 2).

**Table 2:** The statistical differences of CFD expression levels in different clinical stages

Group	H	BGD	I	II	III ˜ IV
	*P*	*P*	*P*	*P*	*P*
**H**	—	> 0.05	< 0.05	< 0.05	< 0.05
**BGD**	> 0.05	—	< 0.05	< 0.05	< 0.05
**I**	< 0.05	< 0.05	—	0.17	< 0.05
**II**	< 0.05	< 0.05	0.17	—	0.74
**III ˜ IV**	< 0.05	< 0.05	< 0.05	0.74	—

Abbreviations: H: healthy controls; BGD: benign gastric disease.

### Discrimination between serum CFD, CEA, CA19- 9, CA72-4 and CA50 in early screening of GC

**Figure 3:**
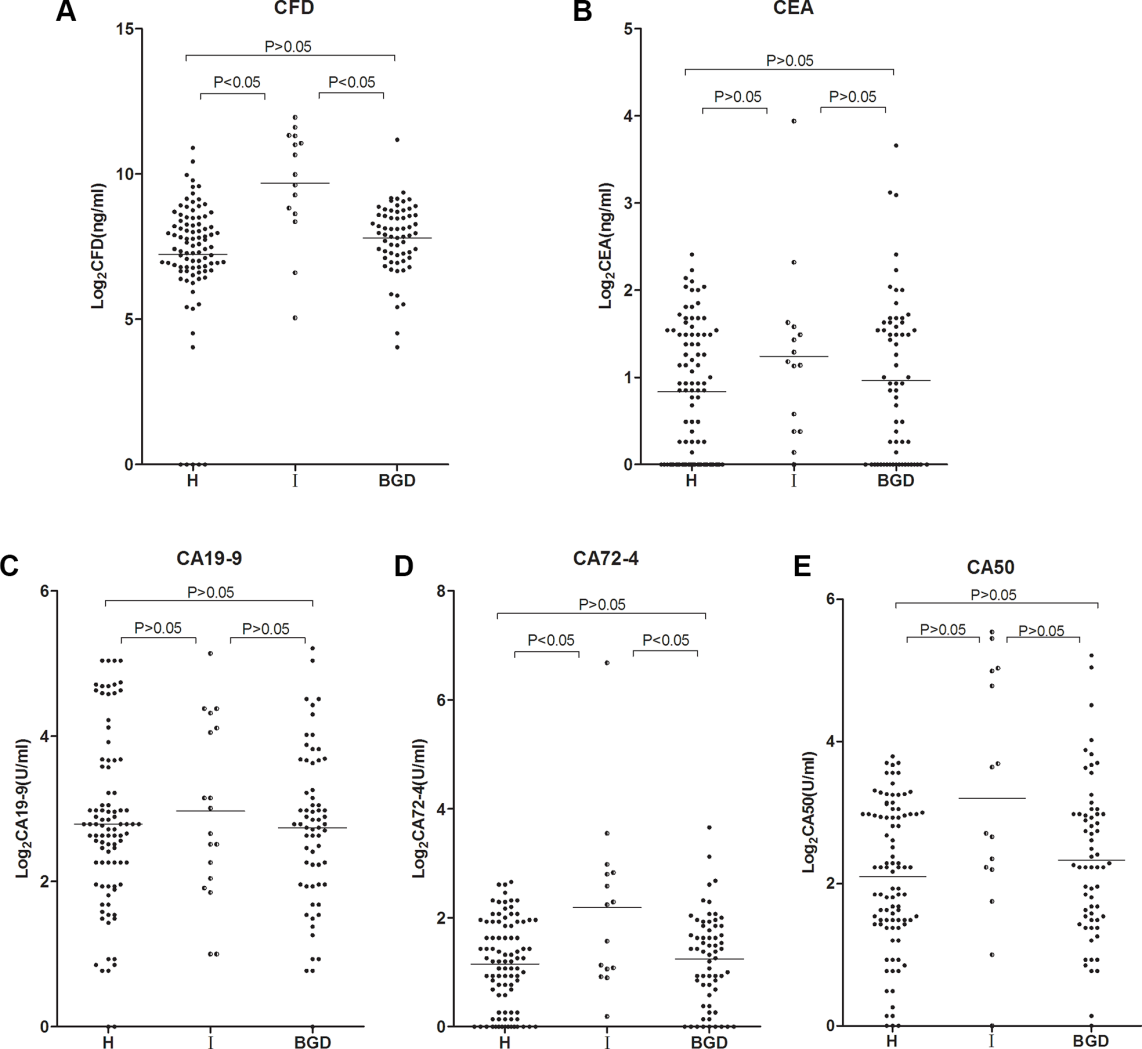
CFD, CEA, CA19-9, CA72-4 and CA50 levels in stage I GC patients, BGD patients and healthy controls Mann Whitney test was used in this figure. CFD, CEA, CA19-9, CA72-4 and CA50 levels were measured in 15 stage I GC patients, 64 unselected BGD patients, and 92 healthy controls. The results for the CFD levels are presented as the median with the 25th and the 75th percentile values. Horizontal lines indicate the median for each group. CFD: cell-free DNA; H: healthy controls; I: stage I of GC patients; BGD: benign gastric disease.

Serum CFD, CEA, CA19-9, CA72-4 and CA50 levels were measured in stage I GC patients, BGD patients and healthy controls to examine their potentiality as GC biomarkers. The CFD is increased at early stage, when other tumor markers have not increased. CFD was detected in all stage I GC samples and its concentration was significantly higher than BGD group and healthy controls (*P* < 0.05) (Figure 3A), but for conventional tumor markers except CA72-4, there was no significant difference in CEA, CA19-9, and CA50 levels between stage I GC patients, BGD patients and healthy controls (Figure 3B–3E).

### Evaluation of CFD, CEA, CA19-9, CA72-4 and CA50 in assistant diagnosis of GC

**Figure 4:**
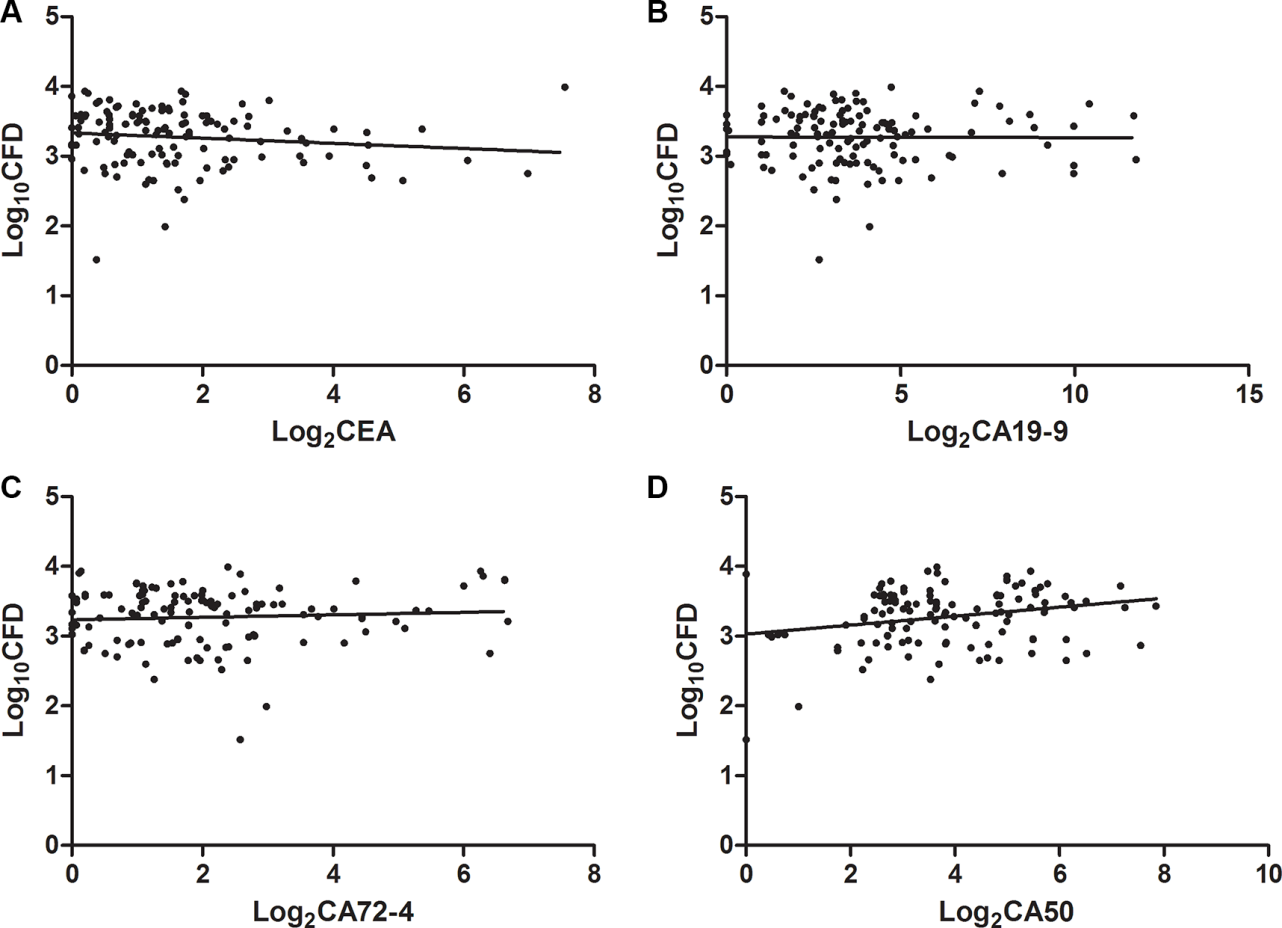
Correlations between CFD concentrations and those of CEA, CA19-9, CA72-4 and CA50 in 124 specimens from GC patients Spearman Correlations was used in this figure. The correlation between CFD levels and CEA, CA19-9, CA72-4 and CA50 in all GC patients (*n* = 124). The data were correlated using linear regression analysis for each biomarker. CEA level (*r* = −0.20, *P* < 0.05, (**A**), CA19-9 level (*r* = −0.06, *P* > 0.05, (**B**), CA72-4 level (*r* = 0.01, *P* > 0.05, (**C**), CA50 level (*r* = 0.21, *P* < 0.05, (**D**). CFD: cell-free DNA; CEA: carcinoembryonic antigen; CA19-9: carbohydrate antigen 19-9; CA72-4: carbohydrate antigen 72-4; CA50: carbohydrate antigen 50.

**Figure 5:**
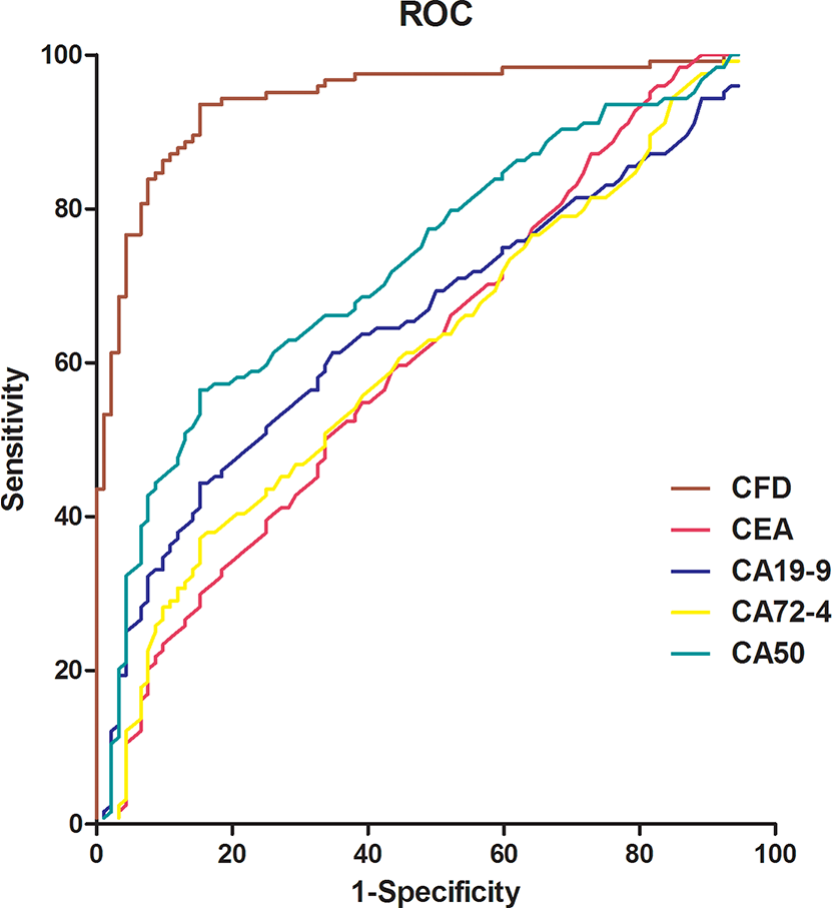
ROC curves for GC diagnosis using serum CFD, CEA, CA19-9, CA72-4 and CA50 ROC curves analysis of *Alu*-based CFD, CEA, CA19-9, CA72-4 and CA50 between GC patients and healthy controls. The AUC was estimated using the logistic procedure in GraphPad Prism v5.0 software, AUC was 0.94 for CFD (95% CI, 0.91–0.97); 0.68 for CEA (95% CI, 0.61–0.75); 0.64 for CA19-9 (95% CI, 0.56–0.71); 0.67 for CA72-4 (95% CI, 0.60–0.74); and 0.81 for CA50 (95% CI, 0.75–0.86). CFD: cell-free DNA; CEA: carcinoembryonic antigen; CA19-9: carbohydrate antigen 19-9; CA72-4: carbohydrate antigen 72–4; CA50: carbohydrate antigen 50.

Spearman correlation analysis was performed to evaluate the potentiality of *Alu*-based CFD as a biomarker. No correlation was observed between *Alu*-based CFD and CA19-9 (*r* = −0.06, *P* > 0.05) or CA72-4 (*r* = 0.01, *P* > 0.05) (Figure 4B, 4C), while there was weak correlation between *Alu*-based CFD and CEA (*r* = −0.20, *P* < 0.05) or CA50 (*r* = 0.21, *P* < 0.05) (Figure 4A, 4D). The ROC curve was plotted to identify a cut-off value that could distinguish between GC patients and healthy controls. The maximal likelihood ratio was 532.70 ng/ml, and this value was chosen as the optimal cut-off. At the optimal cut-off value, the level of *Alu*-based CFD presented a 78.96% sensitivity and 91.81% specificity in separating GC patients from healthy controls with an AUC of 0.94. ROC curves of CEA, CA19-9, CA72-4 and CA50 in GC patients were compared with those in the healthy controls (Figure 5) to screen the optimal diagnostic cut-off value, 95% CI, sensitivity and specificity (Table 3).

**Table 3:** Comparisons between CFD and CEA, CA19-9, CA72-4 and CA50 for GC diagnosis

	AUC	95% CI	*P*	Cut-off	Sensitivity (%)	Specificity (%)
**CFD**	0.94	0.91–0.97	< 0.0001	532.70 ng/ml	78.96	91.81
**CEA**	0.68	0.61–0.75	< 0.0001	5.70 ng/ml	12.50	98.33
**CA19-9**	0.64	0.56–0.71	0.0006	33.10 U/ml	25.70	66.67
**CA72-4**	0.67	0.60–0.74	< 0.0001	6.42 U/ml	27.50	97.62
**CA50**	0.81	0.75–0.86	< 0.0001	13.86 U/ml	50.00	98.81

The data in this table derived from ROC-analysis.

## DISCUSSION

Gastric cancer is the most common gastrointestinal tumor in the world, but early detection remains a challenge in the effective clinical treatment of GC. CFD detection provides a new and simple way for early screening of GC. In the present study, we want to assess the value of CFD in early screening and prognosis of GC.

Detection of CFD in peripheral blood and related molecular biological changes has become a highlight in the research of tumor cell biology and molecular biology in recent years [11, 12]. To date, several techniques have been employed for the quantification of free DNA in the blood, including radioimmunoassays, real-time quantitative PCR, and so on [13–15]. However, as there is an extremely small amount of CFD in peripheral blood at nanogram per milliliter levels, DNA concentration and purity are not enough for traditional detection. Considering the current limitations in CFD detection, the present study was attempted to improve the current detection technology based on branched DNA detection technology by selecting the more sensitive tumor-related genes *Alu* sequence, hoping to find GC, GC recurrence or metastasis in subclinical stages. This signal amplification technology improved detection sensitivity by increasing the labeled probe copy number or signal intensity of the markers without extracting and purifying CFD in peripheral blood, nor amplifying the target sequence, thus overcoming the false positive rate of PCR. In this study, it was found that the CFD content in GC patients was significantly higher than that in healthy controls (*P* < 0.05), which is consistent with the result of CFD detection by Sai et al. [16] and Kolesnikova et al. [17], indicating that the bDNA technology is an ideal method for serum CFD quantitative detection.

Additionally, correlation analysis on the levels of serum CFD and the clinical features revealed that there was no statistically significant correlation between serum DNA concentrations and gender, age, tumor location. As the disease progresses, the levels of serum CFD in stage III ˜ IV GC patients were significantly higher than those in stage I GC patients. Also, CFD levels in GC patients with tumor size > 5 cm were significantly higher than those with tumor size < 5 cm. Czeiger et al. [18] also found that serum CFD concentration was positively correlated with tumor size in their *in vivo* experiment in CD1 mice. These results show that over expression of serum CFD may indicate disease progression and poor prognosis. Further analysis of serum CFD levels and clinical TNM staging showed that the serum CFD concentration in early (stage I) GC patients was significantly higher than that in healthy controls, but except CA72-4 there was no significant elevation in serum CEA, CA19-9 and CA50 levels, suggesting that the elevation of serum CFD is an early event in GC carcinogenesis compared with the traditional tumor biomarkers, and serum CFD detection may be of great significance in early GC screening and diagnosis.

Finally, comparison of serum CFD and CEA, CA19-9, CA72-4, CA50 with ROC curves in the diagnostic efficacy of GC showed that AUC for serum CFD (AUC = 0.94) was significantly greater than that for the other traditional indicators: CEA (AUC = 0.68), CA19-9 (AUC = 0.64), CA72-4 (AUC = 0.67), and CA50 (AUC = 0.81), indicating a higher diagnostic performance, which is consistent with the result of Boni et al. [19] and Frattini et al. [20]. Based on the maximum likelihood ratio of 532.70 ng/mL as the critical value, the level of *Alu*-based CFD presented 78.96% sensitivity and 91.81% specificity in separating GC patients, which are significantly higher than serum CEA, CA19-9, CA72-4 and CA50.

In summary, serum CFD level detection by the bDNA method could serve as an auxiliary tool due to simplicity and high sensitivity. Serum CFD is a more sensitive biomarker for GC patients as compared with serum CEA, CA19-9, CA72-4 and CA50, as well as a more favorable assistant biomarker for early screening of GC.

## MATERIALS AND METHODS

### Patients and sample collection

Included in this study were 124 patients (89 male and 35 female) with primary GC who received treatment at the Departments of General Surgery and Digestive System in the Affiliated Hospital of Nantong University (Nantong, China) between August 2011 and May 2015. The mean age of the patients at the time of diagnosis was 62 years old (range 34–87 years). During the same period, 92 healthy volunteers (60 male and 32 female) who were confirmed to be cancer-free by clinical and imaging examinations were used as negative controls, whose mean age was 49 years old (range 18–78 years). In addition, benign gastric disease (BGD) of 64 patients (29 male and 35 female) had been diagnosed with gastric adenoma were used as positive controls, whose mean age was 56 years old (range 37–82 years). The study protocol was approved by the Institutional Review Boards of the Affiliated Hospital of Nantong University. All data and specimens for the study were collected after obtaining informed consent from the participants.

Peripheral blood (4–5 ml) was collected from each subject in a BE vacuum tube containing separation gel, and sent to the laboratory for immediate processing. The blood samples were separated at 4°C for 2 h and centrifuged at 1600 g for 10 min. The sera were stored at −80°C until use.

### Quantification of CFD

The concentration of CFD was quantitated by bDNA-based *Alu* assay established by Jing RR et al. of our research team [21].

### Quantification of CEA, CA19-9, C72-4 and CA50

CEA, CA19-9, C72-4 and CA50 concentrations were determined by ABBOTT ARCHITECT I2000 SR. The serum levels of CEA, CA19-9, CA72-4 and CA50 were determined by microparticle enzyme (NAME:ARCHITECT CEA, CA19-9, CA72-4 and CA50 Reaget Kit) before operation. A CEA value of > 5 ng/ml, a CA19-9 value of > 37 U/ml, a CA72-4 value of > 7 U/ml, and a CA50 value of > 20 U/ml were considered abnormal.

### Statistical analysis

Statistical analysis was performed with GraphPad Prism v5.0 software. The results for *Alu*, CEA, CA19-9, C72-4 and CA50 concentrations are presented as the median with the 25^th^ and the 75^th^ percentile values. Inter-group comparison of nonparametric quantitative data was performed with the Mann Whitney test. Comparison of nonparametric quantitative data more than two groups was performed with the Kruskal-Wallis test. Correlation was analyzed by using the Spearman test. Receiver operating characteristic curves (ROC) were generated to assess the diagnostic accuracy of each parameter, and the sensitivity and specificity of the optimum cut-off point were defined as values that maximized the AUC 95% was defined as confidence interval (CI) of ROC. All statistical tests were two-sided, and a *P* value of smaller than 0.05 was considered statistically significant.
